# Ultrafast all-optical tuning of direct-gap semiconductor metasurfaces

**DOI:** 10.1038/s41467-017-00019-3

**Published:** 2017-05-12

**Authors:** Maxim R. Shcherbakov, Sheng Liu, Varvara V. Zubyuk, Aleksandr Vaskin, Polina P. Vabishchevich, Gordon Keeler, Thomas Pertsch, Tatyana V. Dolgova, Isabelle Staude, Igal Brener, Andrey A. Fedyanin

**Affiliations:** 1 Faculty of Physics, Lomonosov Moscow State University, Moscow, 119991 Russia; 2 Center for Integrated Nanotechnologies, Sandia National Laboratories, Albuquerque, New Mexico 87185 USA; 3 Institute of Applied Physics, Abbe Center of Photonics, Friedrich Schiller University Jena, Jena, 07743 Germany

## Abstract

Optical metasurfaces are regular quasi-planar nanopatterns that can apply diverse spatial and spectral transformations to light waves. However, metasurfaces are no longer adjustable after fabrication, and a critical challenge is to realise a technique of tuning their optical properties that is both fast and efficient. We experimentally realise an ultrafast tunable metasurface consisting of subwavelength gallium arsenide nanoparticles supporting Mie-type resonances in the near infrared. Using transient reflectance spectroscopy, we demonstrate a picosecond-scale absolute reflectance modulation of up to 0.35 at the magnetic dipole resonance of the metasurfaces and a spectral shift of the resonance by 30 nm, both achieved at unprecedentedly low pump fluences of less than 400 μJ cm^–2^. Our findings thereby enable a versatile tool for ultrafast and efficient control of light using light.

## Abstract (web summary)

Metasurfaces are not adjustable after fabrication, and a critical challenge is to realise a technique of tuning their optical properties that is both fast and efficient. Here, Shcherbakov *et al.* realise an ultrafast tunable metasurface with picosecond-scale large absolute reflectance modulation at low pump fluences.

## Introduction

Optical metasurfaces, which consist of designed subwavelength building blocks arranged in two dimensions, provide a versatile platform for the manipulation of light fields^1–4^. While comprehensive spatial, spectral and polarisation control of the optical response was demonstrated by a large body of research, achieving fast and efficient temporal control remains an open challenge. Such control would dramatically enhance the scope of optical metasurfaces, as their functionalities would no longer be permanently encoded during the fabrication process but could be tuned on demand. Numerous ways of actively modulating the optical properties of metasurfaces were reported, including phase-change materials^5, 6^, mechanical tuning^7, 8^, liquid-crystal-based tuning^9^, and all-optical modulation^10–13^. However, none of these approaches allows for fast and efficient modulation at the same time.

A new generation of tunable metasurfaces was recently enabled by high-index semiconductor materials in their transparency windows^14–18^. The interest in Mie-resonant semiconductor metasurfaces is triggered by their low absorption losses as compared to plasmonic metasurfaces as well as by the diversification of optical engineering options by adding magnetic dipole (MD) Mie-type resonances to the toolbox^19, 20^. Furthermore, semiconductors provide the possibility of modulating the refractive index via the injection of free carriers (FCs); this effect is quite subtle in metals due to the very high initial electron density. Nonlinear-optical properties of semiconductor metasurfaces were recently found to be improved by orders of magnitude when compared with the respective constituent materials^21–26^, paving the way to efficient all-optical tuning.

Silicon has been employed to demonstrate all-optical modulation in Mie-resonant nanostructures^23, 27–29^. Although silicon is the most obvious choice of material for semiconductor metasurfaces, due to its widespread use in microelectronics and on-chip photonics, its indirect bandgap does not allow for efficient generation and recombination of FCs. Gallium arsenide is a material vastly utilised for all-optical modulation; examples include coupled waveguides^30^, microring cavities^31^, and photonic crystal cavities^32, 33^, to name a few. Although there are clear advantages for using direct-gap semiconductors for ultrafast all-optical tuning of metasurfaces, an experimental demonstration of this concept is still missing.

In this work, we report on ultrafast and efficient all-optical tuning of Mie-resonant GaAs metasurfaces. By means of femtosecond wide-band pump–probe spectroscopy, we demonstrate, for the first time to our knowledge, free-carrier-induced absolute reflectance modulation of up to 0.35 under low pump fluence values (not exceeding 400 μJ cm^–2^) and with recovery times of about 6 ps mainly determined by surface-mediated recombination processes. The observed reflectance modulation is explained by ultrafast tuning of the spectral position of the MD resonance by up to ∆*λ* = 30 nm, as well as its broadening, and agrees well with full-wave numerical simulations of the metasurface response that is based on a model for the FC dynamics.

## Results

### Samples

**Fig. 1:**
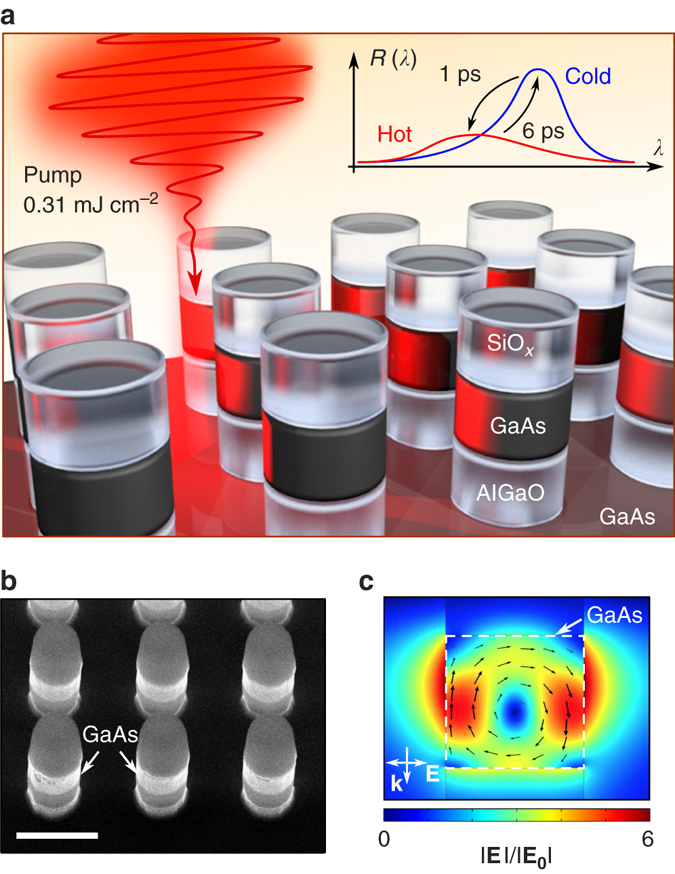
Tuning GaAs metasurfaces with femtosecond laser pulses. **a** Illustration of ultrafast tuning of the MD mode at low pump fluences. The resonance position is tuned within a 6-ps time window due to free carrier injection and subsequent recombination. **b** Scanning electron micrograph of a metasurface sample. The scale bar is 500 nm. **c** Electric fields of the MD mode in the vertical cross-section of a nanodisks, as excited by the probe beam

The device schematic is depicted in Fig. 1a. The GaAs metasurfaces were fabricated using a recently published procedure^34^ that involves mask-etching of GaAs/AlGaAs heterostructures to nanopillars, and subsequent oxidation of an AlGaAs layer to AlGaO. A scanning electron micrograph of a typical sample is shown in Fig. 1b. The residual silica cap on top of the GaAs nanodisk provides for the index matching between the top and the bottom interfaces of the GaAs nanodisks, resulting in more pronounced resonances. The pillars are situated on a bulk GaAs substrate that we used for control measurements. The dimensions of the metasurface are as follows: both diameter and height of GaAs nanodisks are 300 nm, the structure period is 620 nm, the oxide layer thickness is 300 nm, the silica cap thickness is 200 nm. The MD mode of a GaAs nanodisk is visualised in Fig. 1c, where the local electric field map is given; *see* Methods and Supplementary Note 1 for the calculation details. The field amplitude and direction form a vortex-type profile, which is characteristic to the MD resonance in nanodisks^15^.

### Pump–probe spectroscopy

**Fig. 2:**
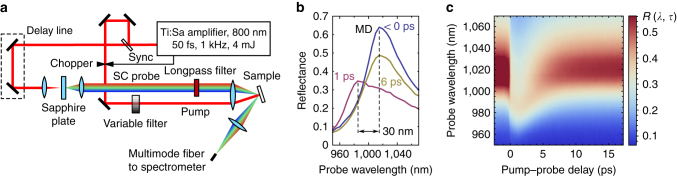
Experimental ultrafast all-optical tuning of GaAs metasurfaces. **a** Setup for broadband pump–probe spectroscopy. Amplified femtosecond pulses are used both for pumping metasurfaces and for generation of the supercontinuum (SC) probe. Transients are measured as a function of both time delay between the pump and probe pulses and probe wavelength. **b** Experimental reflectance of the sample at different pump–probe delays. The MD mode blueshift of 30 nm is observed at a pump fluence of 310 μJ cm^−2^. **c** Transient reflectance spectra that reveal the ultrafast modulation of the resonance within a 6-ps time window. Note that the reflectance values higher than 0.56 are clipped with the false-colour scale for the sake of better visualisation of the post-pump processes

Active ultrafast control over light propagation can be accomplished via femtosecond-pulse-induced FC generation, which is an efficient modulation process utilised, for example, in nonlinear integrated photonics^35, 36^, computational photonics^37^, and terahertz metasurfaces^38^. In order to explore the tuning possibilities in GaAs metasurfaces, we employ pump–probe transient reflectance spectroscopy using supercontinuum radiation as a probe. The setup is schematically shown in Fig. 2a. Amplified femtosecond laser pulses were split into pump and probe beams; the former consisted of a 50-fs, 800-nm-centred, 1-kHz pulse train and the latter was an 850–1300 nm supercoutinuum generated in a sapphire plate and conditioned by a longpass filter. Both beams were focused at the sample plane; the *p*-polarised pump was normally incident, and the *p*-polarised probe was reflected at an angle of 11.5 ± 0.5° from the normal; for more details, refer to Methods.

In our pump–probe measurements, we focus on the MD mode. The MD mode is centred at 1018 nm and has a *Q*-factor of approximately 20, as shown in Fig. 2b with the *blue curve*. In the same panel, the reflectance is shown for two pump–probe delay values of *τ* = 1 ps, where the MD mode is blue-shifted by ∆*λ* = 30 nm due to generated FCs, and *τ* = 6 ps, where its central wavelength position is recovered. This is a situation opposite to that observed in Si nanodisks^28^, where heating was shown to dominate the resonance behaviour, causing it to red-shift. In Fig. 2c, the transient dynamics of the mode is shown as a function of time delay in the range of τ from −2 to 17 ps. The analysis shows that the blue shift is attributed to reduction of the real part of the refractive index of GaAs via the band filling effect and the Drude term^39^:

1 $$\Delta n=\Delta {n}_{{\rm{BF}}}+\Delta {n}_{{\rm{D}}} < 0$$

The detailed analysis of these contributions will be given below.

**Fig. 3:**
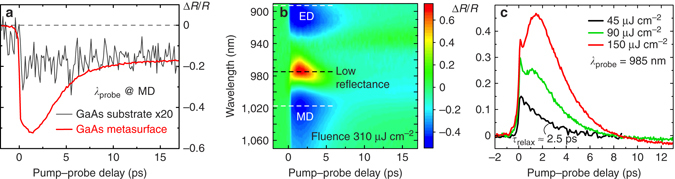
Transient optical properties of GaAs metasurfaces. **a** Transient reflectance measurement results for the sample (*red curve*) and for the substrate (*black curve*; ordinate values are multiplied by 20 for comparison) at a pump fluence of 380 μJ cm^−2^. An enhancement factor of about 50 is attained for the modulation depth at the metasurface sample. **b** Measured differential reflectance as a function of probe wavelength and time delay between the pump and probe pulses. White dashed lines denote the spectral positions of the ED and MD modes, while the black one indicates the area of low reflectance situated in between the resonances. **c** Power-dependent transient reflectance traces. The low-perturbation regime (*black curve*) reveals exponential decay with a time constant of *τ*_relax_ ≈ 2.5 ps, whereas higher fluences (*red and green curves*) provide more complicated traces owing to the blue shift of the MD resonance

The metasurface provides all-optical modulation superior to that of the bulk GaAs. Two typical pump–probe transients are shown in Fig. 3a as recorded for the sample excited at its MD mode (*red curve*) and for the substrate (*black curve*; the modulation values are multiplied by 20 for comparison). We find that the modulation depth is enhanced by a factor of 50 at the metasurface when compared to the substrate. Second, it is apparent that the initially high modulation of the metasurface has a short relaxation time, although there is a residue after 6–7 ps, which will be discussed below. In contrast, the initial relaxation of the substrate happens on the scale of 100 ps. Though an additional discussion is needed to clarify the observations, a subwavelength-scale nanodisk metasurface acts as an efficient active element for all-optical modulation.

A comprehensive study of all-optical modulation as a function of experimental parameters is presented in Fig. 3b,c. Transients were measured as a function of both pump–probe delay and probe wavelength in the spectral vicinity of the electric dipole (ED) and MD resonances. A low-*Q* regime allows for ultrafast cavity buildup time that does not affect the “on”–“off” cycle the way it does in high-*Q* systems like photonic crystal cavities^40^. Fig. 3b reveals both positive and negative all-optical modulation, as well as a variety of relaxation behaviours observed for the metasurface. Even for fluence values as low as 310 μJ cm^–2^, we observe a considerable relative all-optical modulation spanning from ∆*R*/*R* = −50 to 73%, where ∆*R* = *R*_pump_ − *R*, *R* is reflectance in the absence of the pump beam and *R*_pump_ is reflectance in the presence of the pump beam; see Methods for further details. The maximum relative modulation of 90% was achieved at a fluence of 380 μJ cm^–2^ at *λ*_probe_ = 975 nm; further fluence increase brought irreversible changes to the pump–probe traces.

### Relaxation dynamics analysis

On each pump–probe trace, we point out two characteristic periods: a FC-dominated part (0–6 ps) and a phonon-dominated part (>6 ps); the latter is identified by referring to characteristic time scales of the relaxation processes in semiconductors^41^. Here, we will focus on the origins of the ultrafast component, that is, related to FCs, and leave the consideration of the phonon relaxation out of the scope of the paper. After the dense FC plasma is generated, because of the high initial temperature of FCs, thermalisation and cooling of carriers happen within 1 ps^41^. FC temperature dynamics is included in the model describing the band filling term; refer to Supplementary Note 4. Assuming the FC density is *N*, the dynamics of a dense e-h plasma is defined by the following expression^42^:

2 $$\frac{{\rm d}N}{{\rm d}t}=-{{AN}}-{{{BN}}}^{2}-{C}_{{\rm{eff}}}{N}^{3},$$

where *A* is the monomolecular coefficient responsible for radiationless decay through defects and surface states, *B* is the bimolecular recombination with radiation emission, and *C*_eff_ is the effective Auger recombination coefficient that takes into account all the three-body processes, including scattering of excess carriers to the side valleys. We estimate the density of injected FCs as *N*_0_ = 2*F*(1−e^−*αh*^)/*hE*_pump_, where *h* = 300 nm is the metasurface thickness, *α* = 1.4 × 10^4^ cm^–1^ is the GaAs linear absorption coefficient at *E*_pump_ = 1.55 eV, and *F* is the pump fluence. This estimate does not take into account possible absorption increase due to resonances at 800 nm; however, as full-wave simulations show, the metasurface is not resonant at the pump wavelength. While the bulk value of monomolecular relaxation rate is as low as *A*_bulk_ = 7 × 10^7^ s^–1^ (*see* ref. 42) and cannot be connected to the ultrafast relaxation we observe, it was reported repeatedly that nanostructuring affects monomolecular recombination via surface states with energy levels within the band gap^33, 43^. We verify that in our case, in the low-density regime—i.e., at a fluence value of 45 μJ cm^–2^, or estimated plasma density of *N*_0_ ≈ 4·10^18^ cm^–3^—the time constant of the FC recombination process is approximately *τ*_relax_ ≈ 2.5 ps, as derived from the *black curve* in Fig. 3c. At plasma densities this low, the fast relaxation cannot be attributed to high order processes, as the characteristic times obtained through two-body and three-body contributions combined are 340 ps, as calculated by numerically solving Eq. (2); *see* Supplementary Note 2. Therefore, from the *black curve* in Fig. 3c, we can extract the effective monomolecular coefficient, which is *A*_eff_ = 1/*τ*_relax_ ≈ 4.0 × 10^11^ s^–1^. The second-order and third-order recombination processes with *B* = 1.7 × 10^–10^ cm^3^ s^–1^ (*see* ref. 44) and *C*_*e*ff_ = 7 × 10^–30^ cm^6^ s^–1^ (*see* ref. 42) provide a slight reduction of the FC relaxation constant. The overall initial relaxation rate is expressed as follows:

3 $$\frac{1}{\it{\Gamma}}={A}_{{\rm{eff}}}+B{N}_{0}+{C}_{{\rm{eff}}}{N}_{0}^{2},$$

and for the densest initial plasmas of *N*_0_ = 7.5 × 10^19^ cm^–3^ that we estimate from calculations, the second term of the right-hand side is 0.13 of the first, and the third one is 0.10 of the first. Putting all the contributions together, the lowest value of *Γ* is estimated at ≈1.9 ps, with the dominant contribution from the surface recombination processes.

### Refractive index modulation by FCs

Here, we will consider two main mechanisms responsible for the observed tuning of the MD mode: the Drude and band filling terms. The Drude term implies a negative addition to the refractive index^39^:

4 $$\Delta {n}_{{\rm{D}}}(N,E)=-\left(\frac{{N}_{{\rm{e}}}}{{m}_{{\rm{e}}}}+{N}_{{\rm{h}}}\frac{{m}_{{\rm{hh}}}^{0.5}+{m}_{{\rm{lh}}}^{0.5}}{{m}_{{\rm{hh}}}^{1.5}+{m}_{{\rm{lh}}}^{1.5}}\right)\frac{{\hslash }^{2}{e}^{2}}{2{n}{\varepsilon }_{0}({E}^{2}+{\hslash }^{2}{\gamma }^{2})},$$

where *N*_e_ and *N*_h_ are the densities of electrons and holes, respectively (we assume *N*_e_ = *N*_h_ = *N*/2); *m*_e_, *m*_hh_ and *m*_lh_ are the effective masses of electrons, light holes and heavy holes, respectively; *n* is the initial index of GaAs, *γ* is the inverse collision time of the carriers (the damping factor), and *ε*_0_ is the permittivity of vacuum. The band filling term is derived from the FC-modified interband absorption spectrum through the Kramers–Kronig relation:

5 $$\begin{matrix} \Delta \Delta \!{n}_{{\rm{BF}}}(N,E,T)=\frac{2c\hslash }{{e}^{2}}{\rm PV}{\int }_{\!\!\!\!0}^{\infty }\frac{\Delta \alpha (N,E{\prime},T)}{{E^{\prime} }^{2}-{E}^{2}}{\rm d}E{\prime} ,\end{matrix}$$

where ∆*α*(*N*, *E*, *T*) is the modulation of absorption for photon energies *E* lying above the band gap, and “PV” denotes the principal value. The derivation of ∆*α* as a function of *N*, *E* and the FC temperature *T* is given in Supplementary Note 4. Given Eqs. (4) and (5) and ∆*λ *≈ *λ*_0_∆*n*/*n*, the experimental value of ∆*λ* = 30 nm at *τ* = 1 ps can be reproduced using the FC density of *N* = 4.9 × 10^19^ cm^–3^ (the corresponding value at *τ* = 0 ps is *N*_0_ = 7.5 × 10^19^ cm^–3^); the maximum refractive index change is ∆*n* = −0.14.

### Simulations

**Fig. 4:**
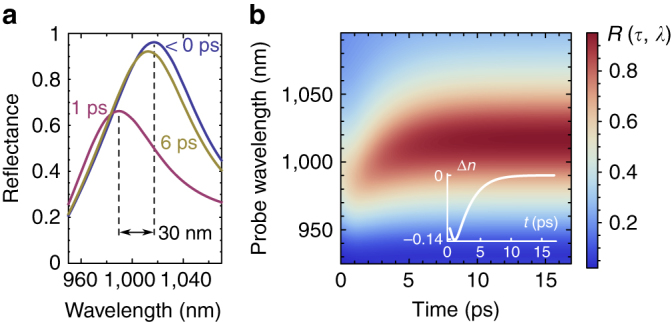
Full-wave simulation results. Transient reflectance spectra obtained using COMSOL Multiphysics with Eqs. (2), (4), and (5) describing the refractive index dynamics. **a** Reflectance of the sample at *τ* < 0 ps (pre-pump, *blue curve*), *τ* = 1 ps (carriers cooled, *purple curve*) and *τ* = 6 ps (carriers recombined, *yellow curve*). **b** Reflectance as a function of probe wavelength and time. The inset shows the time-dependent addition to the index of GaAs used in calculations

In order to verify the experimental data and the physics behind the metasurface tunability, we perform full-wave simulations using COMSOL Multiphysics; see Methods for details. In the simulations, the reflectance spectra of the metasurface are calculated as a function of time with the dielectric function of the GaAs modified according to Eqs. (2), (4), and (5) at the initial plasma density of *N*_0_ = 7.5 × 10^19^ cm^–3^. For better correspondence between the calculations and experiment, we increased the damping factor *γ* of the Drude term by an order of magnitude; this correction might come from an increase of the damping in nanostructures^45^. Calculated reflectance is plotted in Fig. 4 as a function of time and wavelength, along with the corresponding cross sections at times <0, 1 and 6 ps. In the inset of Fig. 4b, the calculated dynamics of the real part of the refractive index is given. The non-exponential behaviour of ∆*n* is in agreement with the experimental data and is attributed to the temperature dependence of the band filling term. With the parameters used, we also observe excellent agreement with the experiment in terms of the MD mode dynamics: 30 nm shift at 1 ps delay and characteristic recovery of the resonance central wavelength in about 6 ps.

## Discussion

Analysing the efficiency of the observed all-optical modulation, it becomes evident that using direct-gap semiconductors as the constituent material for metasurfaces is key to developing active nonlinear-optical nanodevices. Indeed, if compared to indirect-gap materials like silicon, all the FC-related microscopic processes are in favour of direct-gap nanostructures: larger linear absorption and more efficient recombination lead to absolute modulation as high as 0.35 at a fluence of 310 μJ cm^–2^ and relaxation constant *Γ* < 2 ps, whereas in silicon, the most pronounced reported modulation was 0.2 at >1 mJ cm^–2^ (ref. 28) at a 30-ps relaxation. Another recent approach to all-optical modulation in nanostructures is using conductive transparent oxides^46–48^. However, their performance is still limited by high fluences of 1–10 mJ cm^–2^ needed to observe considerable modulation, and material properties that constrain their operation to the near-IR. In contrast, samples based on Mie-type resonances and direct-gap semiconductors can be utilised in the visible with judicious choice of semiconductors. For further comparison of GaAs metasurfaces with other types of metasurfaces—including silicon, metallic, and hybrid approaches,—please refer to Supplementary Note 3. For any subwavelength technology presented in the literature, we find that direct-gap semiconductor metasurfaces presented in our study allow for the most efficient, ultrafast and low-power all-optical modulation solution reported thus far.

To conclude, we have demonstrated an actively tunable metasurface based on ultrafast injection and relaxation of FCs in a direct-gap semiconductor. Arrays of Mie-resonant GaAs nanodisks provide for low-power, efficient and ultrafast all-optical modulation enabled by improved absorption together with rapid recombination likely enabled by surface states. As an outlook, the proposed concept of actively and efficiently tuning the metasurface optical properties may become the basis of a wide class of novel ultrafast metadevices. Application of our approach to metasurfaces may open a range of possibilities for ultrafast wavefront control, including for example beam steering^49, 50^, beam shaping^49, 51^, magnetic mirrors^52^, polarisation manipulation^53^, holography^54^, imaging^55, 56^, and spectroscopy^57^, as well as enabling novel integrated devices. The suggested direct-gap semiconductor metasurfaces may find applications as spatial light modulators with orders-of-magnitude reduced switching times as compared to common liquid-crystal based solutions, and could for example allow ultrahigh bit-rate spatial multiplexing or stimulate the development of new schemes for coherent control of light-matter interactions.

## Methods

### Sample fabrication and characterisation

The GaAs metasurface sample was fabricated starting from metal-organic chemical vapour deposition of a 300-nm thick GaAs layer on top of a 300-nm thick Al_0.85_Ga_0.15_As layer on a semi-insulating GaAs substrate. Etch masks were created using standard electron-beam lithography that converted the exposed negative tone hydrogen silsesquioxane (HSQ Fox-16) to SiO_*x*_. The unexposed HSQ was then developed using tetramethylammonium hydroxide leaving SiO_*x*_ nanodisks as etch masks. The mask shape was transferred onto the GaAs and Al_0.85_Ga_0.15_As layer using inductively coupled-plasma etch. The refractive index isolation between the top GaAs dielectric resonators and lower layers (including the AlGaAs layer and the GaAs substrate) was realized by a selective wet oxidisation process that converts the Al_0.85_Ga_0.15_As into its oxide (Al_*x*_Ga_1–*x*_)_2_O_3_, which has a low refractive index of ≈1.6.

### Pump–probe spectroscopy

Femtosecond laser pulses from a Ti:Sa regenerative amplifier (Coherent Libra USPHE) with 50-fs-long pulses at a photon energy of 1.55 eV and a repetition rate of 1 kHz were used to investigate the ultrafast carrier dynamics in the samples under study. The fundamental pulse with controllably variable average power from 10 to 500 μW (from 0.01 to 0.5 μJ per pulse) was used as a pump. It was focused at the sample into a radius spot with a diameter of ≈350 μm, where a transient carrier distribution in GaAs nanodisks was excited. The probe beam was a white light supercontinuum that was generated in a 4-mm-thick sapphire plate. After a longpass filter, the probe beam covered the spectral range of 850–1300 nm. It had an average power *P* = 0.3 μW and was focused on the sample into the spot with a diameter of ≈80 μm. The time delay between the pump and the probe pulses was varied by a computer-controlled mechanical stage (Sigma Koki OSMS series) in the pump arm. Upon reflection from the perturbed sample, the probe beam was brought to the input slit of a monochromator (Avesta ASP-IR) coupled to an InGaAs photodiode; the spectral resolution was set to 5 nm. The pump beam was chopped at 500 Hz by a trigger-controlled chopper (ThorLabs MC2000B). The pump-induced variation of reflectance was software lock-in detected at the frequency of the optical chopper (*I*_500 Hz_) along with the amplitude of the reflected signal itself (*I*_1 kHz_). However, since the relation *I*_500 Hz_ ≪ *I*_1 kHz_ does not hold, *I*_1 kHz_ is affected by *I*_500 Hz_, having ∆*R*/*R* = *I*_500 Hz_/(*I*_1 kHz_ – *I*_500 kHz_/2). Note that *I*_500 kHz_ is a phase-sensitive signal that reveals both positive and negative transients. The measured data is a series of differential reflectance spectra ∆*R*/*R* for different time delays between pump and probe. A variable optical density filter was used to attenuate the pump beam fluences down to a range of 10–500 μJ cm^–2^. Data in Fig. 2b,c was obtained by combining the measured ∆*R*/*R* spectra and the linear spectra as provided by angle-resolved reflectance spectroscopy.

### Calculations

For the numerical calculation of the transient GaAs metasurface reflectance, we used the commercial software package COMSOL Multiphysics with Floquet periodic boundary conditions and plane wave excitation from the top at an angle of incidence of 12°. Perfect magnetic conductor boundary conditions were used at the symmetry plane to reduce the size of the computational volume by a factor of two. At the GaAs substrate side, a perfectly matched layer mimicking the response of a GaAs half space terminated the computational volume. The dimensions of the structure were as follows: the period of the structure was set to 620 nm, the silica cap was 200 nm in height, the GaAs nanodisk was 300 nm in height, the AlGaO layer was 330 nm in height, and the diameter of the whole pillar was taken to be 236 nm. The discrepancy between the diameter taken from SEM images and the one used in simulations may be due to sample imperfections, such as deviations of the fabricated pillars from a perfect cylindrical shape. For the complex dielectric permittivity function of GaAs we used experimental data from Palik^58^, to which we added the transient response of the induced dense plasma. The AlGaO oxide pedestal and the cap layer were modelled as non-dispersive dielectric media with *n* = 1.6 and *n* = 1.45, respectively.

### Data availability

The data that support the findings of this study are available from the corresponding author upon reasonable request.

## Electronic supplementary material

Supplementary Information Supplementary Figures, Supplementary Notes and Supplementary References
