# Supplementary material for: Ultrafast all-optical tuning of direct-gap semiconductor metasurfaces
Source: Nat Commun. 2017 May 12;8:17. doi: 10.1038/s41467-017-00019-3 (PMC5432034; doi:10.1038/s41467-017-00019-3)
Supplement: Supplementary file 1 — Supplementary Figures, Supplementary Notes and Supplementary References [file 41467_2017_19_MOESM1_ESM.pdf]

Supplementary note 1

Reflectance spectroscopy: experiment and calculation results

Reflectance of the sample was measured using a home-built setup allowing for angular-resolved spectroscopy. The setup consisted of a halogen lamp coupled to a monochromator as a light source, focusing system with NA < 0.1, a Glan polarizer, a goniometer with an XYZ-translating sample holder, and an InGaAs photodiode. The beam output from the monochromator was chopped by an optical chopper at 170 Hz, and the signal from the photodiode was lock-in detected. Recorded values for each wavelength and angle of incidence were divided by the values obtained for a silver mirror. The experimental reflectance spectra are presented in Supplementary Figure 1(a).

For the numerical calculations of the transient GaAs metasurface reflectance we used the commercial software package COMSOL Multiphysics with Floquet periodic boundary conditions and plane wave excitation from the top. Perfect magnetic conductor boundary

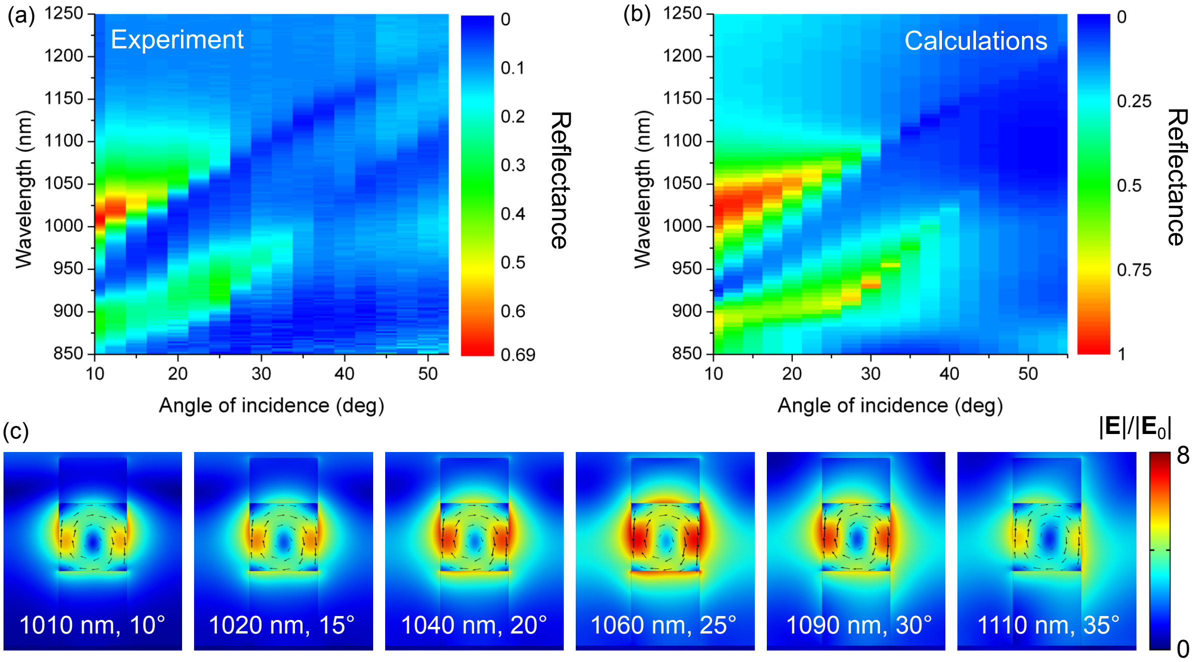

Supplementary Figure 1. (a) Experimental reflectance of the sample under p-polarized infrared light illumination. (b) Corresponding simulation results. (c) Local maps of the normalized electric field strength for the MD mode excited at different angles of incidence. The section of the pillar is taken within the plane of incidence.

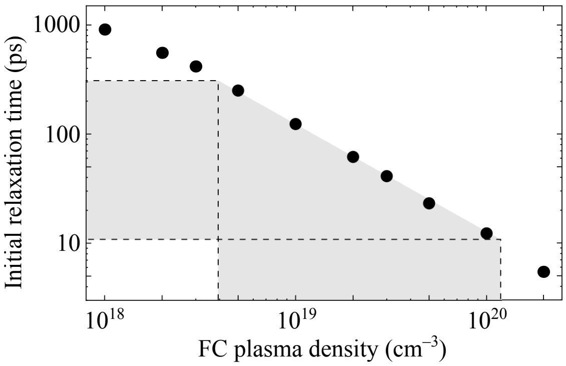

Supplementary Figure 2. Initial relaxation time Γ as a function of the initial FC density in GaAs *without* taking into account surface recombination (black dots). Typical FC densities used in this work and associated relaxation times are marked with a shaded area.

conditions were used at the symmetry plane to reduce the size of the computational volume by a factor of two. At the GaAs substrate side, a perfectly matched layer mimicking the response of a GaAs half space terminated the computational volume. For the complex dielectric permittivity function of GaAs we used experimental data from Palik, to which we added the transient response of the induced dense plasma. The AlGaO pedestal and the cap layer were modelled as a non-dispersive dielectric media with $n = 1.6$ and $n = 1.45$, respectively. The resulting angular-resolved spectra are given in Supplementary Figure 1(b).

Supplementary note 2

Role of two- and three-body recombination in fast relaxation of e-h plasma

Eq.(2) of the main text, which represents the plasma recombination dynamics in GaAs, was solved numerically by Wolfram Mathematica. In order to estimate the possible effect of two- and three-body processes on the relaxation time, we perform calculations of the initial relaxation rate as a function of injected electron-hole plasma density in the *absence* of the enhanced surface recombination, i.e., $A = 0$. Using the known parameters $B = 1.7 \cdot 10^{-10}$ cm^{3}s^{-1} [1] and $C_{\text{eff}} = 7 \cdot 10^{-30}$ cm^{6}s^{-1} [2], one obtains the dependence of Γ on the initial plasma density, as shown in Fig. 2. It can be seen that for the estimated plasma density ranges found in experiments, the fastest high-order-process relaxation is more than 10 ps, while experimentally, at the lowest plasma density, the relaxation was observed to be as fast as 2.5 ps. Therefore, the dominating role of the surface-mediated recombination is established.

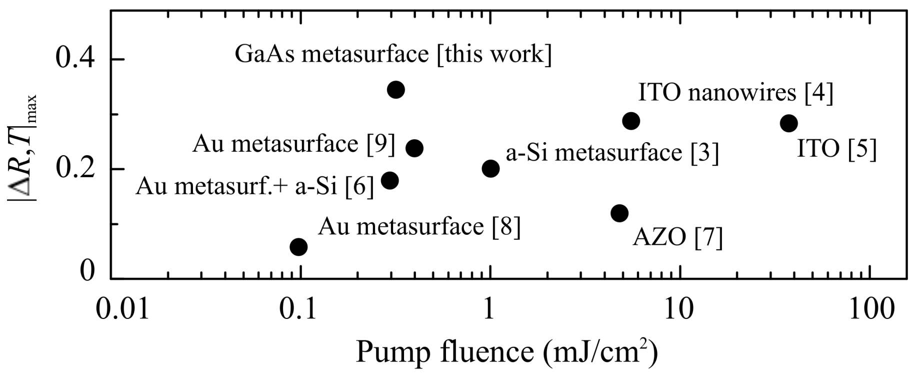

Supplementary Figure 3. Maximum attained ultrafast (< 10 ps) absolute all-optical modulation of light by metasurfaces or thin films as a function of the pump fluence used. Works with the absolute modulation of either transmittance or reflectance of more than 0.05 are shown.

Supplementary note 3

A comparison of all-optical modulation in metasurfaces

In Supplementary Figure 3, a summary of efficiencies for some types of reported metasurfaces is given; only those data are included where the switching cycle is less than 10 ps [3–9]. Maximum attained all-optical modulation is represented by the ratio between the light intensity in "on" and "off" states, either in reflection or transmission. All the presented approaches either are not capable of providing with considerable modulation, or require large optical powers to attain one. On the other hand, in GaAs metasurfaces, due to the direct-gap-assisted light-matter interactions, the all-optical modulation is possible at low fluences, and, at the same time, GaAs provides the most efficient ultrafast all-optical modulation available for subwavelength structures so far.

Supplementary note 4

Dynamics of the refractive index of photoexcited gallium arsenide

Here, we provide the ansatz behind the refractive index dynamics in GaAs metasurfaces. Upon FC injection, the refractive index change relies on three main components [10]: the Drude term, the band filling effect and the band shrinkage effect; the resulting index modulation is given by:

$$\Delta n = \Delta n_{\mathrm{D}} + \Delta n_{\mathrm{BF}} + \Delta n_{\mathrm{BS}}. \tag{1}$$

The Drude term can be expressed as follows:

$$\Delta n_{\mathrm{D}}(N, E) = -\left(\frac{N_{\mathrm{e}}}{m_{\mathrm{e}}} + N_{\mathrm{h}}\frac{m_{\mathrm{hh}}^{0.5} + m_{\mathrm{lh}}^{0.5}}{m_{\mathrm{hh}}^{1.5} + m_{\mathrm{lh}}^{1.5}}\right)\frac{\hbar^2 e^2}{2n_0\varepsilon_0(E^2 + \hbar^2\gamma^2)}, \tag{2}$$

where N_{e} and N_{h} are the densities of electrons and holes, respectively (we assume $N_{\mathrm{e}} = N_{\mathrm{h}} = N/2$); m_{e}, m_{lh}, and m_{hh} are the masses of electrons, light holes and heavy holes, respectively; ε_0 is the permittivity of vacuum, and n_0 is the unperturbed refractive index.

The band filling effect on the refractive index is defined by the decline of the interband transitions due to occupation of the electron and hole states in the conductance and valence bands, respectively. In the parabolic band approximation, the interband absorption is given by the following expression:

$$\alpha(E) = \begin{cases} 0, \quad \text{if} \quad E \leq E_{\mathrm{g}}, \\ \frac{C_{\mathrm{hh}}}{E}\sqrt{E - E_{\mathrm{g}}} + \frac{C_{\mathrm{lh}}}{E}\sqrt{E - E_{\mathrm{g}}}, \quad \text{if} \quad E > E_{\mathrm{g}}, \end{cases} \tag{3}$$

where $C_{\mathrm{hh}} = 3.1 \cdot 10^6$ cm^{-1}eV$^{1/2}$ and $C_{\mathrm{lh}} = 1.6 \cdot 10^6$ cm^{-1}eV$^{1/2}$, and $E_{\mathrm{g}} = 1.42$ eV is the band gap width of GaAs at room temperature. The decrease in interband absorption for photon energies above the band gap is given by:

$$\Delta\alpha = \frac{C_{\mathrm{hh}}}{E}\sqrt{E - E_{\mathrm{g}}}[f_{\mathrm{v}}(E_{\mathrm{ah}}) - f_{\mathrm{c}}(E_{\mathrm{bh}}) - 1] + \frac{C_{\mathrm{lh}}}{E}\sqrt{E - E_{\mathrm{g}}}[f_{\mathrm{v}}(E_{\mathrm{al}}) - f_{\mathrm{c}}(E_{\mathrm{bl}}) - 1], \tag{4}$$

where

$$f_{\mathrm{c}}(E_{\mathrm{bh,bl}}) = \frac{1}{1 + \exp\left(\frac{E_{\mathrm{bh,bl}} - E_{\mathrm{F_c}}}{k_{\mathrm{B}}T}\right)}, \tag{5}$$

and

$$f_{\mathrm{v}}(E_{\mathrm{ah,al}}) = \frac{1}{1 + \exp\left(\frac{E_{\mathrm{ah,al}} - E_{\mathrm{F_v}}}{k_{\mathrm{B}}T}\right)}, \tag{6}$$

are the Fermi-Dirac distributions in for electrons and holes, respectively. The quasi-Fermi levels are given by approximate expressions:

$$E_{\mathrm{Fc}} = \left[\ln\left(\frac{N_{\mathrm{e}}}{N_{\mathrm{c}}}\right) + \frac{N_{\mathrm{e}}}{N_{\mathrm{c}}} \left[64 + 0.05524 \frac{N_{\mathrm{e}}}{N_{\mathrm{c}}} \left(64 + \sqrt{\frac{N_{\mathrm{e}}}{N_{\mathrm{c}}}} \right) \right]^{-1/4} \right] k_{\mathrm{B}} T, \tag{7}$$

and

$$E_{\mathrm{Fv}} = - \left[\ln\left(\frac{N_{\mathrm{p}}}{N_{\mathrm{v}}}\right) + \frac{N_{\mathrm{p}}}{N_{\mathrm{v}}} \left[64 + 0.05524 \frac{N_{\mathrm{p}}}{N_{\mathrm{v}}} \left(64 + \sqrt{\frac{N_{\mathrm{p}}}{N_{\mathrm{v}}}} \right) \right]^{-1/4} \right] k_{\mathrm{B}} T - E_{\mathrm{g}}. \tag{8}$$

Note a misprint in Eq.(8b) of Ref.[10]. N_c and N_v are the densities of states in the conduction and valence bands, respectively:

$$N_{\mathrm{c}} = 2 \left(\frac{m_{\mathrm{e}} k_{\mathrm{B}} T}{2\pi\hbar^2} \right)^{3/2}, \tag{9}$$

$$N_{\mathrm{v}} = 2 \left(\frac{m_{\mathrm{dh}} k_{\mathrm{B}} T}{2\pi\hbar^2} \right)^{3/2}, \tag{10}$$

where $m_{\mathrm{dh}} = (m_{\mathrm{hh}}^{3/2} + m_{\mathrm{lh}}^{3/2})^{2/3}$ is the density-of-states effective mass of holes.

Because of the parabolicity of the bands, the excess energies $E_{\mathrm{ah,al,bh,bl}}$ are directly connected to the excitation energy E through momentum and energy conservation:

$$E_{\mathrm{ah,al}} = (E_{\mathrm{g}} - E) \left(\frac{m_{\mathrm{e}}}{m_{\mathrm{e}} + m_{\mathrm{hh,lh}}} \right) - E_{\mathrm{g}}, \tag{11}$$

$$E_{\mathrm{bh,bl}} = (E - E_{\mathrm{g}}) \left(\frac{m_{\mathrm{hh,lh}}}{m_{\mathrm{e}} + m_{\mathrm{hh,lh}}} \right). \tag{12}$$

We obtain the modulated absorption constant by substituting Eqs.(5–12) into Eq.(4). It is important to note, that, since the injected plasmas are hot, we are interested in Eq.(4) as a function of the non-equilibrium FC temperature. We estimate the average initial temperature to be about $T_{\mathrm{max}} = 15000$ K for the given initial plasma density after Ref. [11]. This value is larger than the initial excess energy of the carriers and, therefore, Fermi-Dirac statistics is not applicable, and our numerical results are an approximation. Thermalization of FCs to this temperature takes place during the first 200 fs after the pump pulse arrives, which is considered as an instantaneous process. Also, the time constant of the carrier-lattice cooling through optical phonon scattering is less than 1 ps given the large initial plasma temperature [12]. The law of plasma cooling is phenomenologically set to be as follows:

$$T(t) = T_{\mathrm{room}} + (T_{\mathrm{max}} - T_{\mathrm{room}}) \exp(-t/\tau_{\mathrm{T}}), \tag{13}$$

where τ_{T} is set to be 0.3 ps.

Having omitted the band shrinkage addition, for its effect on n is more than order of magnitude lower than the other contributions at our plasma densities, one can use the time dependence of $\Delta n(t)$ in order to calculate the reflectance spectra of the metasurface as a function of time. The results are given in Fig. 4 of the main manuscript text.

SUPPLEMENTARY REFERENCES

[1] Y. P. Varshni, "Band-to-band radiative recombination in groups IV, VI, and III-V semiconductors (I)," Phys. Stat. Sol. **19**, 459–514 (1967).

[2] U. Strauss, W. W. Rühle, and K. Köhler, "Auger recombination in intrinsic GaAs," Appl. Phys. Lett. **62**, 55–57 (1993).

[3] M. R. Shcherbakov, P. P. Vabishchevich, A. S. Shorokhov, K. E. Chong, D.-Y. Choi, I. Staude, A. E. Miroshnichenko, D. N. Neshev, A. A. Fedyanin, and Y. S. Kivshar, "Ultrafast all-optical switching with magnetic resonances in nonlinear dielectric nanostructures," Nano Lett. **15**, 6985–6990 (2015).

[4] P. Guo, R. D. Schaller, J. B. Ketterson, and R. P. H. Chang, "Ultrafast switching of tunable infrared plasmons in indium tin oxide nanorod arrays with large absolute amplitude," Nat. Photon. **10**, 267–273 (2016).

[5] M. Z. Alam, I. De Leon, and R. W. Boyd, "Large optical nonlinearity of indium tin oxide in its epsilon-near-zero region," Science **352**, 795–797 (2016).

[6] D. J. Cho, W. Wu, E. Ponizovskaya, P. Chaturvedi, A. M. Bratkovsky, S.-Y. Wang, X. Zhang, F. Wang, and Y. R. Shen, "Ultrafast modulation of optical metamaterials," Opt. Express **17**, 17652–17657 (2009).

[7] N. Kinsey, C. DeVault, J. Kim, M. Ferrera, V. M. Shalaev, and A. Boltasseva, "Epsilon-near-zero Al-doped ZnO for ultrafast switching at telecom wavelengths," Optica **2**, 616–622 (2015).

[8] G. A. Wurtz, R. Pollard, W. Hendren, G. P. Wiederrecht, D. J. Gosztola, V. A. Podolskiy, and A. V. Zayats, "Designed ultrafast optical nonlinearity in a plasmonic nanorod metamaterial enhanced by nonlocality," Nat. Nanotechnol. **6**, 106–110 (2011).

[9] M. Ren, B. Jia, J. Y. Ou, E. Plum, J. Zhang, K. F. MacDonald, A. E. Nikolaenko, J. Xu,

M. Gu, and N. I. Zheludev, “Nanostructured plasmonic medium for terahertz bandwidth all-optical switching,” Adv. Mat. **23**, 5540–5544 (2011).

[10] B. R. Bennett, R. A. Soref, and J. A. Del Alamo, “Carrier-induced change in refractive index of InP, GaAs, and InGaAsP,” IEEE J. Quant. Electron. **26**, 113–122 (1990).

[11] C. V. Shank, R. L. Fork, R. F. Leheny, and J. Shah, “Dynamics of photoexcited GaAs band-edge absorption with subpicosecond resolution,” Phys. Rev. Lett. **42**, 112 (1979).

[12] J. Shah, *Ultrafast Spectroscopy of Semiconductors and Semiconductor Nanostructures*, 2nd ed. (Springer, New York, 1999).
